# Antimicrobial susceptibility profiles, serotype distribution and virulence determinants among invasive, non-invasive and colonizing *Streptococcus agalactiae* (group B streptococcus) from Malaysian patients

**DOI:** 10.1007/s10096-014-2265-x

**Published:** 2014-10-31

**Authors:** N. Eskandarian, Z. Ismail, V. Neela, A. van Belkum, M. N. M. Desa, S. Amin Nordin

**Affiliations:** 1 Department of Medical Microbiology and Parasitology, Faculty of Medicine and Health Sciences, Universiti Putra Malaysia, 43400 Serdang, Selangor Malaysia; 2 Department of Medical Microbiology and Immunology, Faculty of Medicine, Universiti Kebangsaan Malaysia, 56000 Cheras, Kuala Lumpur, Malaysia; 3 bioMérieux R&D Microbiology, La Balme Les Grottes, 38390 France; 4 Department of Biomedical Sciences, Faculty of Medicine and Health Sciences, Universiti Putra Malaysia, 43400 Serdang, Selangor Malaysia

## Abstract

A total of 103 group B streptococci (GBS) including 22 invasive, 21 non-invasive, and 60 colonizing isolates were collected in a Malaysian hospital (June 2010–October 2011). Isolates were characterized by conventional and molecular serotyping and analyzed for *scpB*, *lmb*, *hylB*, *cylE*, *bac*, *bca* and *rib* gene content. Antimicrobial susceptibility to penicillins, macrolides, lincosamides, quinolones and tetracyclines was determined using disk diffusion and the MICs for penicillin were determined by E-test. Molecular serotyping for all eight serotypes (Ia, Ib, II–VII) was in full accordance with conventional serotyping. Overall, taking CS and MS together, serotype VI was the most common capsular type (22.3 %) followed by VII (21.4 %), III (20.4 %), Ia (17.5 %), V (9.7 %), II (7.7 %) and IV (1 %). Susceptibility to beta-lactam antimicrobials was prevalent (100 %). Resistance rates for erythromycin, clindamycin and tetracycline were 23.3 %, 17.5 % and 71.8 %, respectively. PCR-virulence gene screening showed the presence of *cylE*, *lmb*, *scpB* and *hylB* in almost all the isolates while *rib*, *bca*, and *bac* genes were found in 29.1 %, 14.6 % and 9.7 % of the isolates. Certain genes were significantly associated with specific serotypes, namely, *rib* with serotypes Ia, II, III and VI; *bca* and *bac* with serotypes II and III. Furthermore, serotype Ia was significantly more common among patients with invasive infections (*p* < 0.01) and serotype VI isolates were significantly more common among carriers (*p* < 0.05). In summary, serotype distribution correlates with virulence gene content will be useful in epidemiological studies and design of vaccines.

## Introduction

*Streptococcus agalactiae*, the group B streptococcus (GBS), is a gram-positive and encapsulated bacterium, which displays beta-hemolytic activity on blood agar. It is part of the commensal flora in the genital and lower gastrointestinal tracts in 10–40 % of healthy adults [1]. However, vaginal colonization by GBS during pregnancy may be clinically significant because it is associated with neonatal meningitis and septicemia [2]. During recent decades, GBS infection has increasingly emerged in adults, particularly those with underlying illness such as diabetes [3].

Penicillin is the first-line agent for prevention and treatment of GBS infections [1, 4]; however, since 1994, GBS isolates with reduced susceptibility to penicillin have been reported periodically [5]. In case of penicillin allergy, clindamycin and erythromycin are the most recommended alternatives. Consequently, the increasing use of these drugs, consumed by around 20 % of all GBS carriers, resulted in elevated rates of clindamycin and erythromycin resistance worldwide [6, 7]. Capsular serotype contributes to disease severity and defines vaccine development [8]. Ten GBS capsular types have been identified (Ia, Ib and II-IX) and vaccination against five serotypes (Ia, Ib, II, III and V) is in development [7, 9]. Several investigations revealed that strains harboring the CPS types Ia, II, III, and V are the most significant causes of GBS invasive disease in neonates and other patients [10, 11]. PCR can identify serologically non-typable strains since these generally still carry the required genes [12]. Therefore, molecular serotyping techniques have an elevated discriminatory power for epidemiological studies [13].

GBS has many virulence factors including surface proteins, toxins and hydrolytic enzymes [14]. Surface proteins mostly function as adhesins, which may also contribute to immune evasion. These include C5a peptidase (ScpB), laminin-binding protein (Lmb), the α and β-subunits of C protein (Bca and Bac) and Rib protein. GBS secrete a variety of toxins such as β-hemolysin/cytolysin (cylE), hyaluronidase (hylE) and the CAMP factor (cfb) which facilitate entry of GBS into host cells and promote its intra-cellular survival [15].

Epidemiological studies on GBS, assessment of their antibiotic susceptibility and conventional serotyping are sparse in Malaysia. Molecular serotyping and virulence determinants of Malaysian GBS are published for the first time in this study.

## Materials and methods

### Bacterial isolates

A total of 103 pure cultures of GBS were obtained from outpatients and inpatients at the Universiti Kebangsaan Malaysia Medical Centre (UKMMC), a 900-bed teaching and referral hospital in Malaysia (June 2010–October 2011). Isolates were grouped into three different types based on the clinical outcome and site of isolation: invasive (*n* =22) for isolates derived from normally sterile body site such as blood; non-invasive isolates (*n* =21) for isolates derived from wound and skin ulcers, tracheal and gastric aspirates; colonizing isolates (*n* =60) derived from vaginal swabs and urine samples from asymptomatic patients. Basically, pure GBS cultures were obtained except for 10 (9.7 %) cases in which GBS coexisted with other organisms (e.g. *Staphylococcus aureus*, coagulase-negative staphylococci, *Escherichia coli*, *Pseudomonas aeruginosa*, *Klebsiella* species or unspecified gram positive and negative cocci). Identification was carried out based on culture characteristics, Gram staining, catalase testing and CAMP testing. The species nature of the isolates was confirmed by slide agglutination using Streptex (Murex Diagnostics, UK) and by conventional PCR assays targeting the *cfb* gene (encoding the CAMP factor). All strains were preserved in sterile nutrient broth with 15 % glycerol and on beads in cryo-vials (Pro-Lab, UK) at −70 °C for long-term storage and on 5 % sheep blood agar plates at 4 °C for short-term maintenance.

### Serotyping

Capsular serotyping was performed by the latex agglutination method using antisera specific for each capsular type (Ia, Ib, II-VII) using Strep-B-Latex kits (Statens Serum Institute, SSI, Sweden). Isolates that did not react with any of the antisera were classified as serologically non-typeable (NT). Further confirmation of capsular types was performed for all strains using PCR. The conventional PCR assay as developed for seven GBS capsular types (Ia-VII) and targeting the *cps* gene cluster was used according to Borchardt et al. [16]. DNA was extracted using GeneAll Exgene Mini genomic extraction kits (GeneAll, South Korea) following the manufacturer’s instructions. The GBS reference strains A909 (type Ia), H36B (type Ib), 18RS21 (type II), M78 (type III), CNCTC 1/82 (type IV), BAA-611 (type V), NT6 (type VI), 7271 SSI (type VII) were kindly provided by Universiti Kebangsaan Malaysia Medical Centre.

### Antimicrobial susceptibility testing

Antibiotic susceptibility testing (AST) was performed by the disc diffusion (Kirby-Bauer) method according to the guidelines described by CLSI in 2012 [17]. The panel of antibiotics included penicillins (penicillin G and ampicillin), cephalosporins (cefuroxime and ceftriaxone), a quinolone (levofloxacin), a glycopeptide (vancomycin), a macrolide (erythromycin), a lincosamide (clindamycin), a phenicole (chloramphenicol) and tetracycline. The MIC for penicillin was determined using the Etest® strip (bioMérieux, Marcy l’Etoile, France). Each batch of tests was run concurrently with *Streptococcus pneumoniae* ATCC 49619 as quality control. The diameters of the inhibition zones and MICs were interpreted based on CLSI interpretation guidelines (2012) [17]. Erythromycin resistant, clindamycin sensitive strains were further tested by the double-disc diffusion method (D-zone test) with erythromycin and clindamycin disks 12 mm apart. Strains with D-shaped clindamycin zones (inducible clindamycin resistance) were considered as “clindamycin resistant”.

### Determination of virulence genes

All GBS isolates were screened for the presence of seven virulence genes, namely, *scpB*, *lmb*, *hylB*, *cylE*, *bac*, *bca* and *rib* encoding C5a peptidase, laminin binding protein, hyaluronate lyase, β-hemolysin/cytolysin, β-C protein, α-C protein and Rib protein by using PCR. The oligonucleotide primers and PCR cycling conditions were as reported by others [18–21]. The GBS reference strains, ATCC 13813 (*cfb*), ATCC BAA-611 (*rib*), NCTC 11078 (*bca*, and *bac*), ATCC 12403 (*cylE*), ATCC 12386 (*lmb* and *scpB*) and *Streptococcus agalactiae*, strain 4755 (*hylB*), were included as positive controls.

### Statistical analysis

The SPSS statistical analysis package (version 21.0 for Windows) was used to analyze the significance of the results. Chi-square tests of independence were performed to assess possible associations between the variables. The significance level was set at *p* <0.05. If the chi-square test of independence was statistically significant, then post-hoc comparison tests were conducted to determine the variable that produced the statistically most significant difference. The obtained standard residual (std.res) from post-hoc analysis was considered significant when std.res >1.96, >2.58 and >3.33 were at 0.05, 0.01 and 0.001 level of significance.

## Results

Clinical data of all patients are summarized in Table 1. Among the 103 GBS isolates, 11 (10.7 %) were from neonates, all of whom presented with early-onset disease (<7 days old) and more than half of the neonates (64 %) were known to have GBS bacteremia. Ninety-two isolates (89.3 %) were recovered from adults (age range 20–87). In non-pregnant adults (*n* =43), GBS isolates were often isolated from blood (37.3 %), vagina (35 %), wound and abscess specimens (26 %). Urine sample yielded only one isolate (1.7 %). Among pregnant women, most of the GBS isolates were recovered from vaginal specimens (90 %). Blood, urine and placenta swabs yielded a few additional isolates (10 %).

**Table 1:** Distribution of 103 GBS isolates among patient groups

Patient group	No. (%) of patients	Invasive population	Non-invasive population	Colonizing population	Total
Neonate	11 (10.7)	7	4	0	11
Pregnant adults	49 (47.6)	0	3	46	49
Non-pregnant adults	43 (41.7)	15	14	14	43
Total	103 (100)	22	21	60	103

### Comparison of conventional serotyping (CS) with molecular serotyping (MS)

CS was able to identify the capsular serotypes of 80.6 % (83/103) of the GBS isolates studied and 20 (19.4 %) isolates remained non-typable. All GBS isolates were also tested by MS in order to confirm the accuracy of CS. MS was in accordance with CS for the CS-typeable isolates. MS was able to detect capsular serotype genes in all 20 (19.4 %) serologically non-typable isolates. Six isolates showed serotype III while the remaining 14 isolates were of serotype VII. Overall, taking CS and MS together, serotype VI was the most common capsular type (*n* =23, 22.3 %) followed by VII (*n* =22, 21.4 %), III (*n* =21, 20.4 %), Ia (*n* =18, 17.5 %), V (*n* =10, 9.7 %), II (*n* =8, 7.7 %) and IV (*n* =1, 1 %). No serotype Ib isolates were found in the present study.

### Antimicrobial susceptibility profiles

All GBS strains were susceptible to penicillin G, ampicilin, cefuroxime, ceftriaxone, levofloxacin, vancomycin and chloramphenicol. A hundred percent susceptibility to penicillin was also confirmed by E-test where the MIC ranged from 0.023 to 0.064 μg/ml. The AST results showed that the majority of isolates (*n* =74, 71.8 %) were resistant to tetracycline. Twenty-four isolates (23.3 %) were resistant to erythromycin and 18 (17.5 %) to clindamycin. Only one case of the 18 resistant isolates showed inducible clindamycin resistance.

### PCR amplification of the virulence genes

All isolates were positive for the *cfb* gene, which helped to confirm the species identity of GBS. Genes encoding the ***β***-hemolysin/cytolysin (***β***H/C), laminin binding protein, C5a peptidase, and hyaluronate lyase (*cylE*, *lmb*, *scpB* and *hylB*) were present in 97.1 %, 97.1 %, 96.1 % and 94.2 % of isolates, respectively, making them the predominant virulence genes. The *rib*, *bca*, and *bac* genes were found in 29.1 %, 14.6 % and 9.7 % of the isolates, respectively.

### Association of genotypic traits with molecular serotyping

The distribution of three genotypic traits (*bac*, *bca*, *rib*) and various serotypes are presented in Table 2. As *cylE*, *lmb*, *scpB*, *hylB* were detected in almost all GBS isolates (97.1 %, 97.1 %, 96.1 %, 94.2 %, respectively), these were excluded from the analysis. Serotype IV was detected only once and was also excluded. The *rib* gene is significantly associated with serotype Ia, VI (*p* < 0.05), II and III (*p* < 0.01). The *bca* gene was more common among serotype II (*p* < 0.001) and III (*p* < 0.01) strains. A significant association was also found between the *bac* gene and serotype II (*p* < 0.001) and III (*p* < 0.05) (Table 2).

**Table 2:** Association between capsular serotype and virulence genes in 103 isolates of group B streptococcus

Serotype	*rib* ^+^	*p*-value	*bca* ^+^	*p*-value	*bac* ^+^	*p*-value
Ia	1 (5.0 %)	< 0.05	0 (0.0 %)	> 0.05	0 (0.0 %)	> 0.05
II	7 (87.5 %)	< 0.01	7 (87.5 %)	< 0.001	5 (62.5 %)	< 0.001
III	12 (63.2)	< 0.01	8 (42.1 %)	< 0.001	5 (26.3 %)	< 0.01
V	3 (37.5 %)	> 0.05	0 (0.0 %)	> 0.05	0 (0.0 %)	> 0.05
VI	2 (7.7 %)	< 0.05	0 (0.0 %)	> 0.05	0 (0.0 %)	> 0.05
VII	5 (23.8 %)	> 0.05	0 (0.0 %)	> 0.05	0 (0.0 %)	> 0.05
Total	30 (29.4)		15 (14.7 %)		10 (9.8 %)	

*rib* + , *bca* + and *bac*+ indicates presence of *rib*, *bca* and *bac* genes

std. res >1.96, *p* < 0.05

std. res >2.58, *p* < 0.01

std. res >3.33, *p* < 0.001

Although all serotypes were observed among the three populations studied, serotype Ia was significantly more common among patients with invasive infections (*p* < 0.01) and serotype VI isolates were significantly more common among carriers (*p* < 0.05) (Table 3).

**Table 3:** Distribution of the serotypes within the studied GBS populations

Serotype	Invasive population	Non-invasive population	Colonizing population
Ia	10 (45.5)**	6 (28.6)	2 (3.4)
II	1 (4.5)	0 (0.0)	7 (11.8)
III	2 (9.1)	4 (19.0)	15 (25.0)
V	1 (4.5)	2 (9.5)	7 (11.8)
VI	3 (13.7)	3 (14.3)	17 (30.0)*
VII	5 (22.7)	6 (28.6)	11 (18.0)

*p < 0.05, **p < 0.01

No statistically significant associations were found between the virulence genes and the isolate status or age of patients in the present study (*p* >0.05).

## Discussion

In Malaysia, data on detection of GBS virulence genes and molecular serotyping are non-existent. In the present research, GBS isolates were serotyped by both phenotypic and genotypic approaches and further characterized for their virulence patterns and antibiotic susceptibility profiles.

Serotype distribution of GBS isolates has previously been reported to vary geographically [9, 22]. In the present study, MS results showed serotype VI (22.2 %) to be overrepresented in the Malaysian population. In contrast, an older study found lower numbers of serotype VI isolates among adults and neonates [23]. Serotype VII is rarely reported worldwide [7, 24, 25]; however, we found a relatively high number of strains with serotype VII (*n* =22, 21.3 %) among Malaysians. Only eight GBS isolates from our study were CPS type II while a cross-sectional study in South East Asia reported this type as predominant among pregnant women [22]. Serotype Ia was significantly more common among patients with invasive infections (*p* < 0.01) and serotype VI isolates were significantly more common among carriers (*p* < 0.05). All isolates were typable by MS, while a considerable proportion (19.4 %) of isolates could not be typed by CS, as also observed in previous studies [9, 26, 27].

The overall beta-lactam susceptibility of Malaysian GBS was confirmed here [1, 4, 22]. Neither penicillin-resistant nor penicillin intermediate GBS strains were detected. Some investigations have identified GBS isolates with reduced sensitivity [5, 28, 29]. Hence, penicillin could still be used as a first-line drug for intra-partum prophylaxis and treatment of GBS infections in Malaysia.

The rates of resistance to erythromycin and clindamycin were lower than those in the studies from Japan, Europe and the United States [30, 31], but can be considered high in comparison to the studies from Asian countries [32, 33]. The resistance documented for erythromycin and clindamycin provides a warning that these drugs need to be prescribed with care since also non-susceptible GBS were observed in the United States and Japan [28, 34].

The frequency of virulence gene *rib* presence is similar to other studies from Europe, the United States and Lebanon [15, 18, 35] and higher than those reported among pregnant women in Poland and Kuwait [14, 36]. The *bca* encodes ***α***-C surface protein (ACP) was observed in a lower frequency than in previous reports, where its presence varied from 42 to 85 % [15, 36, 37]. Another study has identified the *bca* gene in 100 % of strains; however, in this case only potentially clonal type Ia strains were tested [38]. The *bac* gene was found at a lower frequency (9.7 %) as compared to other studies from the United States, Europe and New Zealand [7, 18, 39]. A recent study among pregnant women in Kuwait detected *bac* in only 3.2 % of isolates [37].

Certain virulence genes were significantly more prevalent in given CPS types. For instance, *rib* was associated significantly with serotypes Ia, II, III and VI. Previous data from South East Asian countries and Europe confirmed the association between *rib* gene and serotype Ia, II and III [39–42]. Moreover, our results indicated a significant association between serotypes II and III with *bca* and *bac*, while other studies demonstrated association between *bca* and *bac* with serotype Ib and II [35, 39, 41]. According to Duarte et al. (2005) around 30–55 % of serotype Ia GBS isolates in humans harbored the *bca* gene [20]. In another study reported by Dore et al. around 50 % of serotype III isolates harbored the *bca* or *bac* or both [42]. This difference may be caused by geographical differences in prevalence or may indicate the appearance of silent *bca* and *bac* genes in these serotypes.

The present study could not demonstrate any correlation between the virulence genes and clinical status of the patients from whom the isolates were obtained (*p* >0.05); however, as pointed out by Maning et al. invasive isolates showed a tendency to have *rib* and *bac* [18]. No significant associations were found between the occurrence of virulence genes and the age of patients in the present study (*p* >0.05). However, based on data presented by Ho et al. the majority of *bca* genes (85.4 %) in GBS isolates were observed in strains from adults, whereas 59.2 % of *rib* gene was harbored among isolates from neonates [40]. Given the lack of correlation we describe above, our data do not confirm the virulence potential of the genes detected.

In conclusion, our GBS collection contained a high number of strains with serotypes VI and VII, in contrast to findings in other countries [7, 24, 43]. Such differences in the relative regional incidence of serotypes could compromise the efficacy of vaccine. Additionally, the present study clearly shows the equivalence of MS and CS and indicates the broader coverage of MS. Data presented here confirms previous findings on the relationship between different virulence genes and serotypes but fails to confirm the virulence contribution of the panel of potential virulence genes we screened for.
